# Temperature Effects on Bacterial Phytochrome

**DOI:** 10.1371/journal.pone.0109794

**Published:** 2014-10-07

**Authors:** Ibrahim Njimona, Rui Yang, Tilman Lamparter

**Affiliations:** Karlsruhe Institute of Technology KIT, Botanical Institute, Karlsruhe, Germany; University of Texas at Austin, United States of America

## Abstract

Bacteriophytochromes (BphPs) are light-sensing regulatory proteins encoded in photosynthetic and non-photosynthetic bacteria. This protein class incorporate bilin as their chromophore, with majority of them bearing a light- regulated His kinase or His kinase related module in the C-terminal. We studied the His kinase actives in the temperature range of 5°C to 40°C on two BphPs, Agp1 from *Agrobacterium tumefaciens* and Cph1 from cyanobacterium *Synechocystis* PCC 6803. As reported, the phosphorylation activities of the far red (FR) irradiated form of the holoprotein is stronger than that of the red (R) irradiated form in both phytochromes. We observed for the apoprotein and FR irradiated holoprotein of Agp1 an increase in the phosphorylation activities from 5°C to 25°C and a decrease from 25°C to 40°C. At 5°C the activities of the apoprotein were significantly lower than those of the FR irradiated holoprotein, which was opposite at 40°C. A similar temperature pattern was observed for Cph1, but the maximum of the apoprotein was at 20°C while the maximum of the FR irradiated holoprotein was at 10°C. At 40°C, prolonged R irradiation leads to an irreversible bleaching of Cph1, an effect which depends on the C-terminal His kinase module. A more prominent and reversible temperature effect on spectral properties of Agp1, mediated by the His kinase, has been reported before. His kinases in phytochromes could therefore share similar temperature characteristics. We also found that phytochrome B mutants of *Arabidopsis* have reduced hypocotyl growth at 37°C in darkness, suggesting that this phytochrome senses the temperature or mediates signal transduction of temperature effects.

## Introduction

Histidine kinases (HKs) are sensory homodimeric proteins which commonly consist of an N-terminal sensory module and a C-terminal effector module. The C-terminal effector module is composed of the dimerization/histidine phosphotransfer (DHp) and catalytic/ATP-binding (CA) domains. HKs are known to catalyze three distinct phosphotransfer reactions: autophosphorylation of the histidine substrate within the DHp domain, and transphosphorylation as well as dephosphorylation of the cognate response regulator (RR). These proteins are essential signal carriers in plants and microorganisms [1], [2]. Bacteria and fungi respond to transient environments through transmembrane integrated HKs, which act in concert with their RRs to elicit necessary adaptive responses that are critical for their survival and virulence [3], [4]. The HKs and RRs have evolved as two-component signal transduction systems (TCS), thereby stimulation of the HK leads to autophosphorylation at a conserved histidine residue which initiates a signaling cascade [5]. In the prototypical two-component pathway, the phosphoryl group is transferred from the HK directly to the RR. In the case of hybrid HKs, the phosphoryl group is first transferred intramolecularly to an aspartate in the receiver domain of the same polypeptide. A histidine transferase then shuttles the phosphoryl group to a soluble RR. The net difference between phosphorylation and dephosphorylation of RR accounts for the quick biological response either by altering the transcriptional level of specific downstream target genes or by direct modulation of molecular motors [6]–[8]. Because of wide prevalence in bacteria and fungi and the absence in humans, TCSs have been considered attractive targets for the development of new potential drugs to control bacterial and fungal infections [9].

Phytochromes are widely distributed photochromic photoreceptors found in bacteria, plants and fungi. These bilin proteins are sensitive in the red and far red region of the visible spectrum [10]. The N-terminal photosensory chromophore (PCM) module is composed of a PAS (Period/Arnt/Single minded), GAF (cGMP phosphodiesterases/Adenylate cyclase/FhlA) and PHY (phytochrome) domain. In fungal and bacterial phytochromes including Agp1 from *Agrobacterium tumefaciens*, a biliverdin chromophore is covalently attached to a conserved Cys at the N-terminal of the PAS domain [11], [12], whereas a conserved Cys in the GAF domain is used by plant- and various cyanobacterial phytochromes including Cph1 from *Synechocystis* PCC 6803 [13]–[16] that use phytochromobilin and phycocyanobilin as chromophore, respectively [17]. Phytochromes are synthesized in the red absorbing form (Pr) which upon absorption of red light photoconverts to the far red absorbing form (Pfr). Pfr converts back to Pr upon irradiation with far red. Besides photoconversion, Pfr to Pr and Pr to Pfr dark conversion have been found for different types of phytochromes. Photoconversion is coupled to protein structural changes [18]–[21] which result in a modulation of signal transduction.

The C-terminus of plant, fungal and most bacterial phytochromes shows significant homology to histidine kinases [22]. The substrate histidine in the H-box of the protein is highly conserved in bacterial and fungal phytochromes, but missing in plant phytochromes with the exception of monocotyledonous PhyA [23]–[25]. Bacterial and fungal phytochromes act as bona fide HKs in which photoconversion alters their kinase activity [26]. In cyanobacterial Cph1 and in Agp1 from *A. tumefaciens*, the bacterial phytochromes used in the present study, autophosphorylation is strong in the Pr and weaker in the Pfr form [18], [26], [27].

Crystal X-ray diffraction and cryo-EM studies show that bacterial phytochromes are homodimeric proteins in which the two subunits are arranged in a parallel manner [28], [29]. The GAF domain forms most of the contacts with the chromophore. The PAS and GAF domains are connected in a knotted structure with the PHY domain forming a tongue-like structure that folds back on the GAF domain and the chromophore, providing a short connection between chromophore and HK [13], [30]–[32]. The PHY domain is shown to stabilize the Pfr form of Cph1 and Agp1 [13], [19] and is connected to the C-terminal HK by a long helix [28]. Intramolecular signal transduction is probably mediated through this connection.

In Agp1, the autophosphorylation shows an unexpected temperature dependency. The strongest phosphorylation signal was obtained at 25°C. With increasing temperature the phosphorylation decreased and was almost undetactable at 40°C, although the cells are still growing at this temperature. Spectral properties of Agp1 are affected by the temperature in a way that had not yet been described for other phytochromes: at 40°C, continuous red irradiation leads to photoconversion of Pfr into another bleached form termed Prx. This temperature effect is dependent on the presence of the HK [33]. These findings led to the suggestion that Agp1 might act as a temperature sensor. A similar loss of phosphorylation at 40°C has been described for the VirA HK of *A. tumefaciens*, which is responsible for activation of virulence genes [34].

In the present study, we extended our studies on Agp1 to a broader temperature range and investigated HK temperature effects of Cph1, a well characterized cyanobacterial phytochrome. Whereas the temperature optimum of Agp1 was between 20°C and 25°C, the Cph1 holoprotein had a temperature optimum at 10°C. At elevated temperature, prolonged irradiation leads to an irreversible bleaching of Cph1, an effect again dependent on the HK.

## Materials and Methods

### Protein expression, purification and assembly

All expression vectors used in these studies encode protein with a C-terminal hexahistidine tag for Ni^2+^ affinity purification. The vectors are based on the pQE12 (Qiagen) expression vector. The cloning of pAG1 and pF10, expression vectors (for full-length Agp1 and Cph1), is described in [27], [35] and the cloning of expression vectors for the truncated proteins lacking the HK is described in [36], [37]. *Escherichia coli* XL1-blue cells with the desired expression vector were grown in 2 L lysogeny broth medium with 0.3 µM final concentration of ampicillin and tetracycline at 37°C until an optical density at 600 nm (OD_600_) of 0.6 was reached. Specific protein expression was induced by adding isopropyl-1-thiol-ß-D-galactopyranoside to a final concentration of 50 µM. After 40 hours incubation at 18°C, the OD_600_ reached 2.3. At this point, the cells were collected and centrifuged at 5000 g for 10 min and the pellet was suspended in 20 mL of extraction buffer (300 mM NaCl, 50 mM Tris/Cl, 5 mM EDTA, pH 7.8) with freshly added 2 mM (final concentration) ß-mercaptoethanole. The cells were lysed with a French Pressure Cell (Aminco) with two passages at 1380 bar. The lysed cells were centrifuged at 20 000 g for 30 min. The soluble proteins in the supernatant were precipitated with 50% ammonium sulfate, and the pellet dissolved in dilute imidazole buffer (300 mM NaCl, 50 mM Tris/HCl, 10 mM imidazole, pH 7.8). The sample was centrifuged again and the supernatant loaded to a 3 cm×5 cm Ni^2+^ affinity chromatography column (Qiagen, Hilden Germany) that was equilibrated with dilute imidazole buffer. The column was then washed with this buffer until non-bound proteins were washed out. The apoprotein was eluted with concentrated imidazole buffer (300 mM NaCl, 50 mM Tris/Cl, 250 mM imidazole, pH 7.8). The eluted protein was pooled and precipitated with 50% ammonium sulfate. The resulting pellet was suspended in extraction buffer and centrifuged again. The supernatant was used for later steps. All purification steps were done at 4°C. The apoprotein concentration was estimated by absorption at 280 nm [27]. For details on the expression and purification of apo-Agp1 and apo-Cph1 see previous publications [18], [27]. For chromophore assembly, 50 µM of DTT and biliverdin (Frontier Scientific) or phycocyanobilin [35] to a final concentration of 60 µM were added to 20 µM protein. The sample was incubated in darkness at 20°C until assembly was complete as monitored by UV/visible photometry. Excess chromophore was separated from the holoprotein using NAP-10 desalting columns (GE Healthcare) as described by the manufacturer. The final buffer for spectral assays was 300 mM NaCl, 50 mM Tris/Cl, 5 mM EDTA, pH 7.8. The pH, adjusted at 4°C, varies with the temperature and was 7.0 at 40°C. Therefore, control measurements were undertaken to test spectra and phosphorylation at 20°C between pH 7 and 8. We found no pH effects on these features that could explain the changes obtained upon temperature change.

### Spectral assays

Absorption spectra were measured in a JASCO V-550 photometer with temperature control unit and a custom-built computer-controlled irradiation device. The scan speed was set to 1000 nm min^−1^; scans were measured between 900 nm and 250 nm. For photoconversion of Cph1, 730 nm far red and 655 nm red light emitting diodes were used. Far red (500 µmol m^–2^ s^–1^) was typically given for 120 s. Red (400 µmol m^–2^ s^–1^) was either given for 60 s of for prolonged times as indicated in the text.

### Kinase assays at different temperatures

For autophosphorylation, an earlier protocol [18], [33] was adopted. Phosphorylation experiments of the holoproteins were performed under blue-green safelight using 505 nm light emitting diodes or in darkness. The protein concentration was 12 µM. Each sample contained 5 µl Agp1 or Cph1, irradiated with either 655 nm red light from a light emitting diode (20 µmol m^−2^ s^−1^) or with 780 nm far red from a light emitting diode (80 µmol m^−2^ s^−1^). The irradiation time was always 2 min, and the temperature during this irradiation was 25°C. Directly after irradiation, 15 µl phosphorylation buffer (final concentrations 25 mM Tris/HCl, 5 mM MgCl_2_, 4 mM β-ME, 0.2 mM EDTA pH 7.8, 50 mM KCl, 5% ethylene glycol, 0.45 µM (50 MBq/ml) γ^−32^P ATP, pH 7.8) were added to each sample. After mixing, the samples were immediately transferred to a thermal block, which was pre-set to a given temperature. The incubation time was 30 min. Thereafter, the phosphorylation reaction was stopped by adding 10 µl of loading buffer (30% glycerol, 6% SDS, 300 mM DTT, 0.01% bromphenol blue, 240 mM Tris/HCl, pH 6.7) to each reaction mixture. Then, 10 µl of each sample were loaded an SDS-PAGE gel (10% acrylamide in separating gel). After electrophoresis, the protein was transferred onto a PVDF membrane (Millipore) with a Trans-Blot semi-dry blot apparatus (Bio-Rad). The dried membrane was exposed to a phosphoimager plate (Fuji) for about 5 min, followed by quantification using the fluorescent image analyzer FLA 2000 (Fuji), and integrated analysis software. The protein on the PVDF membrane was stained with Simply Blue safestain (Invitrogen). Apoprotein phosphorylation experiments were performed in the same manner as for the holoprotein except that the protein was not irradiated and that all incubation steps were under artificial light. Direct comparisons of apo- and holoprotein Agp1 at 25°C revealed a 1.2±0.1 apoprotein/holoprotein (FR irradiated) ratio. This ratio was used to normalize the temperature dependent activities of holo- and apoprotein in Agp1, which were probed in different sets of experiments. The kinase activity of Cph1 was assayed in a similar manner. The ratio of apoprotein/holoprotein (Pr) phosphorylation at 20°C was 0.97±0.1.

### Arabidopsis hypocotyl growth

*Arabidopsis thalinia* phytochrome B mutants N6217 (Col ecotype) and N6218 (Ws ecotype) were obtained from the European Arabidopsis Stock Center Nottingham and propagated in the Botanical Garden. Seeds were surface sterilized for 1 min in 70% EtOH, for 7 min in 1∶1 (v/v) sodium hypochlorite, rinsed 4 times with sterile H_2_0, and placed on growth medium consisting of 1/2 Murashige and Skoog salts [38], 0.05% MES, 0.8% (w/v) agar without sucrose. The plates were incubated for 2 days at 4°C in darkness. Germination was induced by applying 2 hours white light at 23°C. Thereafter, seeds were taken back to darkness for 22 h at 23°C. Germinated seeds were then incubated at different temperatures as given in the text for 48 h on vertical petri dishes. All treatments are done in green safelight or darkness. Thereafter images were taken and hypocotyl lengths were measured using Image J.

## Results

**Figure 1:**
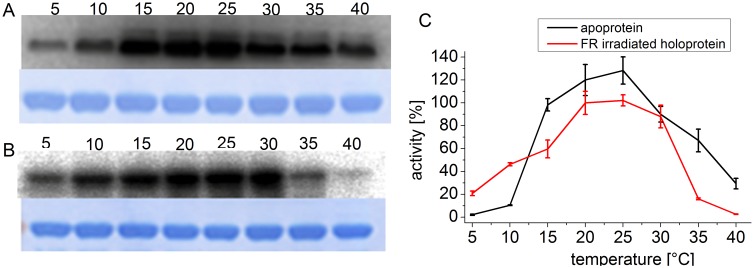
Autophosphorylation of Agp1 at various temperatures. Autoradiogram (above) and Coomassie-stained blot (below) of Agp1 apoprotein (A) and FR irradiated holoprotein (B). The incubation temperature during the phosphorylation assay in °C is given above each lane. (C) Mean phosphorylation intensities of three experiments ± SE as shown in (A) and (B) are plotted over temperature. The 100% value corresponds to the mean signal of the holoprotein at 25°C.

The previous measurements on Agp1 were performed at temperatures of 25°C and above [33]. In order to find the temperature optimum of phosphorylation, we expanded the temperature range for phosphorylation studies. As shown in Fig. 1, the temperature optimum for the apoprotein and the holoprotein is at 20°C to 25°C. As before, the autophosphorylation of the apoprotein at 40°C was still around 20% of the activity at 25°C, but negligible in the holoprotein. Autophosphorylation of the holoprotein at 5°C was still reasonably high, around 20% of the 25°C level. Thus, the order between holo and apoprotein is reverted at low temperature.

A decrease of phosphorylation activity with increasing temperature has also been described for other HK-like VirA and DesK [34], [39]. We therefore asked whether other bacterial phytochromes follow the same pattern. We performed similar experiments with the cyanobacterial phytochrome Cph1 from *Synechocystis* PCC 6803. Apo- and holoprotein were again investigated in separate experiments to keep the number of samples in each experiment in a range that can be handled. In a third series, the phosphorylation of the holoprotein in the Pr and Pfr forms were compared.

**Figure 2:**
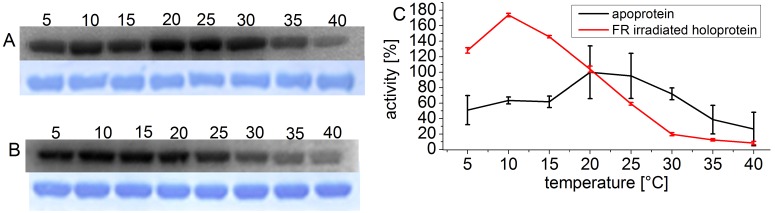
Autophosphorylation of Cph1 at various temperatures. Autoradiogram (above) and Coomassie-stained blot (below) of Cph1 apoprotein (A) and far red light irradiated holoprotein (B). Holoprotein was irradiated with FR and incubated with γ^−32^P ATP in darkness at the indicated temperatures. (C) Mean phosphorylation intensities of three experiments ± SE as shown in (A) and (B) are plotted over temperature. The 100% value corresponds to the mean signal of the holoprotein at 20°C.

**Figure 3:**
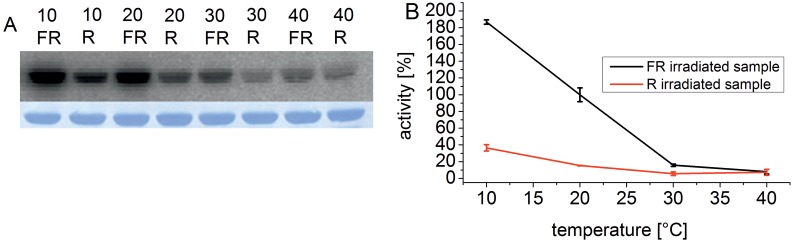
Autophosphorylation of Cph1 at various temperatures. (A) Autoradiogram (above) and Coomassie-stained blot (below) of Cph1 holoprotein. Samples were irradiated either with FR or R as indicated and incubated with γ^−32^P ATP in darkness at the indicated temperatures (°C). (B) Mean phosphorylation intensities of three experiments ± SE as shown in (A) are plotted over temperature. The 100% value corresponds to the mean signal of the FR irradiated sample at 20°C. as shown in (A).

The temperature maximum of the Cph1 holoprotein in the Pr form was at 10°C, and at 5°C the activity was still 80% of the highest value (Fig. 2). At 40°C, the activity was less than 5%. The temperature optimum of the apoprotein was at 20–25°C. The chromophore has thus also a significant impact on the temperature behavior of Cph1 phosphorylation. At 10°C, the activity of the holoprotein is approximately 2.5 fold higher than that of the apoprotein (Fig. 2). With increasing temperature, the ratio between far-red- and red-irradiated holoprotein samples (predominately Pr and Pfr, respectively) diminished (Fig. 3), as in Agp1. Under our measuring conditions this ratio was 5∶1 at 10°C, but almost 1∶1 at 40°C.

### Spectral properties

**Figure 4:**
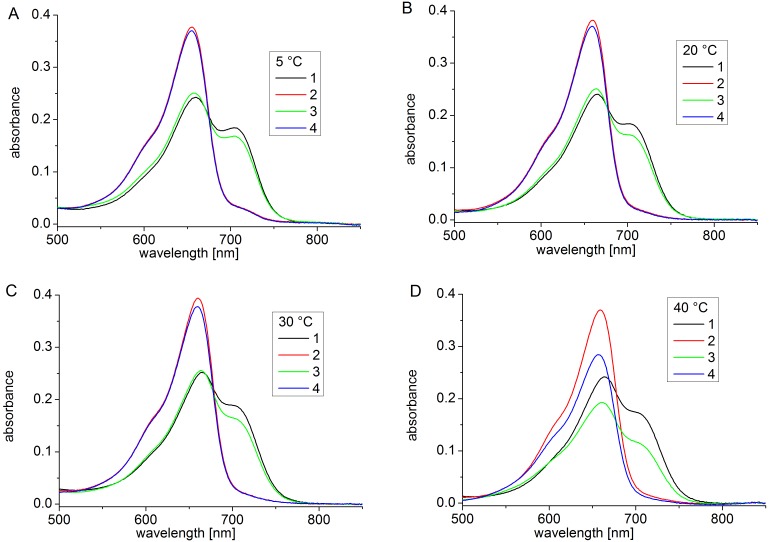
Spectra of full length Cph1 at 5°C (A), 20°C (B), 30°C (C) and 40°C (D). Shown are measurements after temperature adaptation and 2 min R irradiation (1, black lines), after a subsequent FR irradiation (2, red lines), a long (2 h) R irradiation (3, green lines) and a subsequent FR irradiation (4, blue lines). The R light intensity was 400 µmol m^−2^ s^−1^.

**Figure 5:**
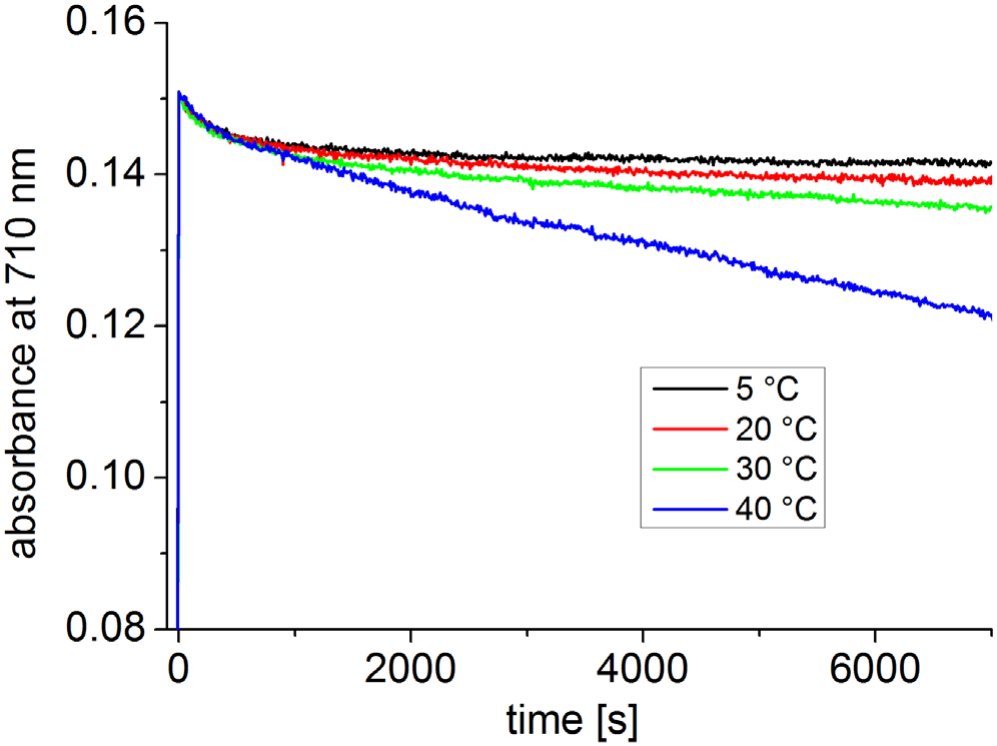
Time drive measurements with full length Cph1. Absorbance at 710 nm (the absorbance maximum of Pfr) was measured continuously; at t = 0 s, R (400 µmol m^−2^ s^−1^) was switched on. The protein concentration of this sample differed slightly from that in Fig. 4.

**Figure 6:**
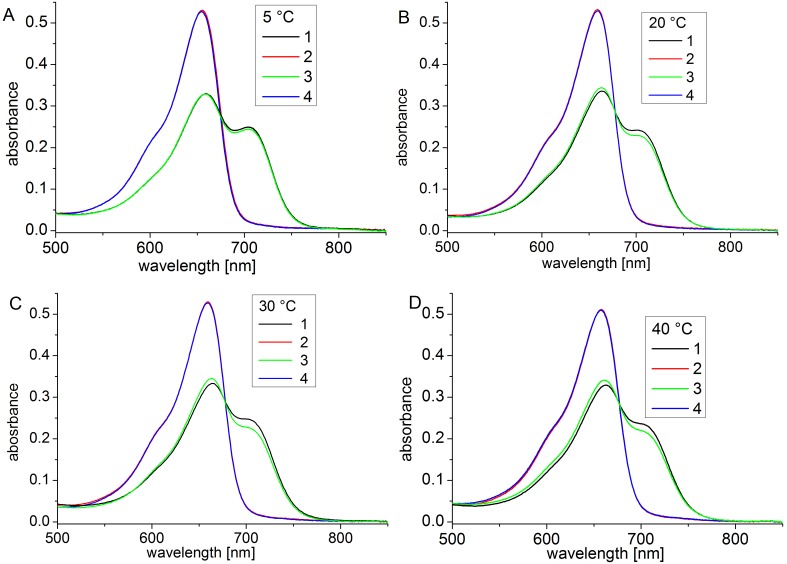
Spectra of the N-terminal PCM of Cph1 at 5°C (A), 20°C (B), 30°C (C) and 40°C (D). Shown are spectra after temperature adaptation and 2 min R irradiation (1, black lines), after a subsequent FR irradiation (2, red lines), a long (2 h) R irradiation (3, green lines) and a subsequent FR irradiation (4, blue lines). The R light intensity was 400 µmol m^−2^ s^−1^.

**Figure 7:**
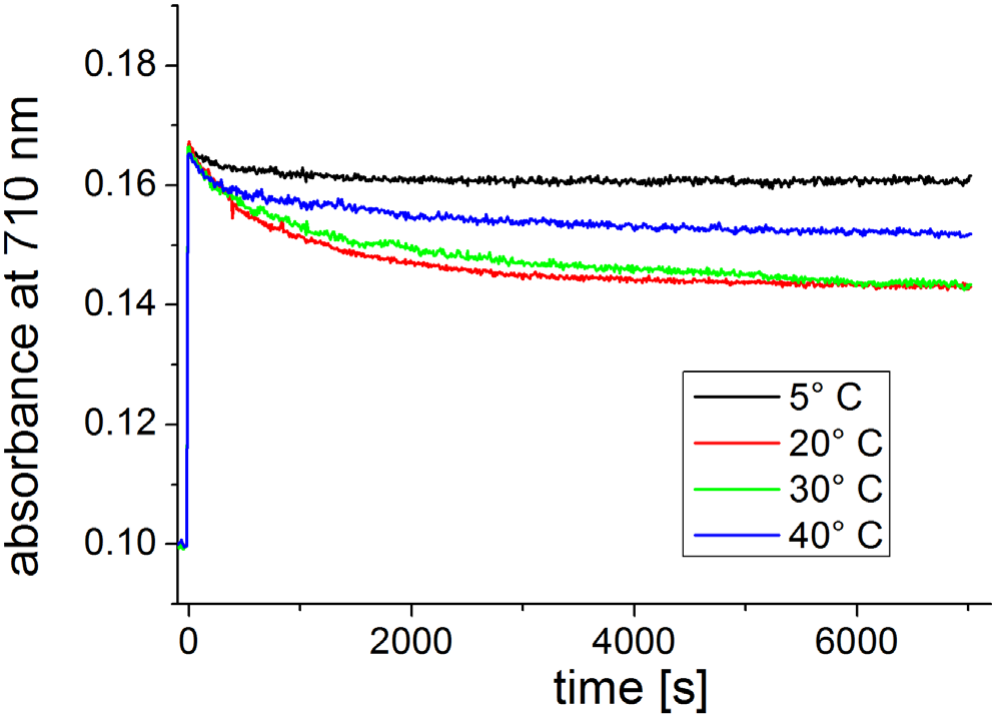
Time drive measurements with PCM of Cph1. Absorbance at 710 nm (the absorbance maximum of Pfr) was measured continuously; at t = 0 s, R (400 µmol m^−2^ s^−1^) was switched on. The protein concentration of this sample differed from that in Fig. 6.

At 35°C and 40°C, Agp1 acquires unusual spectral properties. Continuous irradiation results in the conversion of Pfr into a new spectral species with a decreased extinction coefficient, termed Prx, which might have a deprotonated chromophore. The effect is only found in the presence of the HK [33]. Here, we tested the effect of continuous irradiation on Cph1 at different temperatures. Spectra were taken after FR and R irradiations at 5°C, 20°C, 30°C and 40°C. In a typical setting, Cph1 reference spectra were first taken after 2 min R and after 2 min FR. The samples were then irradiated for 2 h with R. One spectrum was recorded after this treatment and a final one after a 2 min FR irradiation. Spectra and absorbance changes as measured during the 2 h R irradiation period are shown in Figures 4 and 5 for the full length protein. For the full length protein we observed a slight (less than 5%) loss of absorbance at 710 nm upon continued irradiation at 5°C, 20°C, and 30°C (Fig. 5) that affects both Pr and Pfr (Fig. 4). This effect results probably from bleaching of a sensitive subpopulation of Cph1 under the high light intensities (400 µmol m^−2^ s^−1^). Heterogeneity of Cph1 has been demonstrated by various methods [40], [41]. When the samples were irradiated continuously with red light, the absorbance at 710 nm decreased steadily. At 40°C, prolonged irradiation resulted in a continuous loss of absorbance in the range of the Pfr absorbance maximum, which reached approximately 20% after 2 h (Fig. 5). This loss affected both Pr and Pfr absorbance (Fig. 4D). There were no indications for denaturation of the protein during these experiments: denaturation is accompanied by aggregation and increase of scattering, which was not found. When irradiated Cph1 was brought back from 40°C to 20°C, the absorbance did not recover (data not shown). Opposed to Agp1, the temperature effect on the spectra is therefore not reversible. Spectra and absorbance changes of the N-terminal PCM fragment are shown in Figs. 6 and 7, respectively. The spectra recorded at 40°C did not significantly differ from those at the other temperatures. There was a weak bleaching effect at all temperatures which was strongest at 30°C (Fig. 7). Under continuous illumination with red light, the absorbance at 710 nm decreased slightly during the initial hour but did not change thereafter (Figs. 6 and 7). The temperature effect is thus dependent on the presence of the His kinase.

### In vivo temperature effect

**Figure 8:**
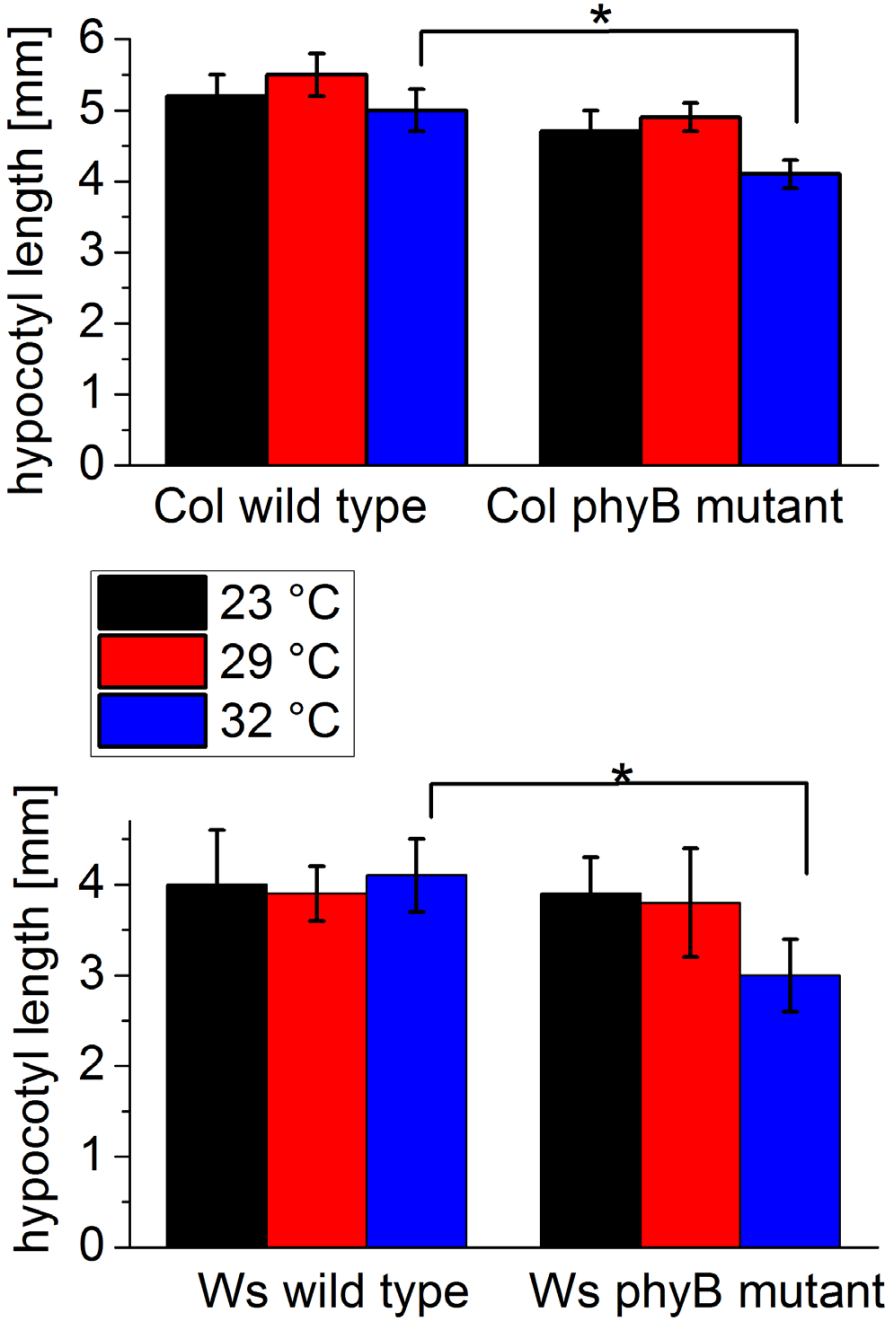
Hypocotyl lengths of Arabidopsis wild type and mutants grown at different temperature in darkness. Mean values ± SE.

In order to see whether there could be a temperature effect of Agp1 in *A. tumefaciens*, we performed growth and swimming plate assays of wild type and knockout mutants but we could not find an effect that would be significantly different between the wild type and the *agp1*- mutant (Njimona, Rottwinkel, Lamparter, unpublished results). The biological effects of Cph1 or Agp1 are not known [42]. For this reason an *in* *vivo* effect of temperature mediated by these phytochromes might be hard to find. We thus performed a temperature experiment with seedlings of *Arabidopsis thaliana*, the model plant for phytochrome responses. The hypocotyl growth of seedlings is inhibited by light, mediated by phytochromes and cryptochromes [43]. We compared hypocotyl lengths of seedlings grown at different temperatures between two different *phyB* mutants and the corresponding wild types Columbia (Col) and Wassilevskia (Ws). In order to avoid interference with light, we performed these growth experiments in darkness only. In both wild type seedlings, no significant difference was found between hypocotyls grown at 23°C, 29°C or 32°C, whereas both *phyB* mutants had shorter hypocotyls at 32°C when compared with the corresponding wild type (Fig. 8). This difference was significant with an error probability of <5%. The differences between wild type and mutant at other temperatures were not significant. The fact that two independent mutants had a similar phenotype indicates that the result is not related to a second site mutation. Phytochrome B is thus required for proper hypocotyl growth at elevated temperature in darkness.

## Discussion

In the ecosystem, organisms have to withstand to fluctuations in temperature on a daily basis. Previous studies have shown that photoreceptors may have a unique role in reacting to environmental temperature change [44]. For phytochromes a thermometer function has not been described. However, most phytochromes have a C-terminal His kinase or His kinase related region which could function as a temperature sensor. Here, we investigated the effect of temperature on the HK activities of two model bacteriophytochromes, Agp1 and Cph1 and on the hypocotyl growth of *Arabidopsis*.

In several cases, HKs act as temperature sensors, either as their sole function, exemplified by Hik33 of *Synechocysti*s PCC 6803 [45] or DesK from *Bacillus subtilis*, [46] or combined with sensing chemical factors as in VirA of *Agrobacterium tumefaciens* [34]. All proteins shows high autophosphorylation activity at 25°C and low activity at 37°C. Recently, Mironov et al. suggested a dual function of Hik33 as red light regulation of cold responses in *Synechocystis* PCC6803 [47], [48]. In the bacteriophytochrome Agp1, a temperature increase above 25°C results in a reduction of HK activity. An even more pronounced effect with an optimum at 10°C was found for the Cph1 holoprotein from *Synechocystis*. It might well be that a decrease of kinase activity with increasing temperature is universal for bacterial phytochromes and other HKs. Both Agp1 and Cph1 also have in common that elevated temperature changed their spectral properties in a HK dependent manner. Phytochrome fragments with PAS and GAF domains but missing PHY domains have photoproduct spectra [18], [19], [49] similar to those of full length Agp1 and Cph1 at elevated temperature ([33] and present work). Elevated temperature could result in partial unfolding of the PHY domain, mediated through the HK that is linked to the C-terminus of the PHY domain. This could lead to disturbance of the chromophore hydrogen bonding network and deprotonation of the chromophore. A third feature common to Cph1 and Agp1 is the combined effect of chromophore abundance and temperature on HK activity. In both proteins the apoprotein kinase activity was stronger than that of the holoprotein at elevated temperature, but weaker in the low temperature range.

Several links between phytochromes and temperature effects have been reported. The phytochrome from *P. aeruginosa* seems to be involved in stress responses and quorum sensing; heat tolerance was impaired in a phytochrome knockout mutant but not in the wild type [50]. In seed plants, phytochromes are important for temperature adaptations [51], with seed germination providing a classic example [52], [53]. In *A. thaliana*, which has five phytochromes (phyA to phyE), the effect of temperature on seed germination has been investigated in wild-type plants and in double and triple *phyA*, *phyB,* and *phyE* mutants. The germination/temperature pattern differed from the wild-type pattern in most mutants (Heschel et al. 2007). The *HFR1* and *PIF4* transcription factors play a central role in temperature adaptation of *A. thaliana.* In *hfr1*, *pif4*, and *phyB* mutants temperature effects on hypocotyl elongation have been investigated [54]. These temperature responses differed from the wild type, in line with a function of phyB as temperature sensor. The question whether the *phyB* mutant is different from the wild type in darkness was not investigated [54]. Our data showed that dark grown *phyB* mutants have a reduced hypocotyl at 32°C as compared to the wild type. These results suggests that phyB acts in the Pr form and plays a role in temperature adaptation; it could itself act as a thermometer.
